# No Impact of Stochastic Galvanic Vestibular Stimulation on Arterial Pressure and Heart Rate Variability in the Elderly Population

**DOI:** 10.3389/fnhum.2021.646127

**Published:** 2021-02-17

**Authors:** Akiyoshi Matsugi, Koji Nagino, Tomoyuki Shiozaki, Yohei Okada, Nobuhiko Mori, Junji Nakamura, Shinya Douchi, Kosuke Oku, Kiyoshi Nagano, Yoshiki Tamaru

**Affiliations:** ^1^Faculty of Rehabilitation, Shijonawate Gakuen University, Osaka, Japan; ^2^Faculty of Allied Health Sciences, Kansai University of Welfare Sciences, Osaka, Japan; ^3^Department of Otolaryngology-Head and Neck Surgery, Nara Medical University, Nara, Japan; ^4^Faculty of Health Science, Kio University, Nara, Japan; ^5^Graduate School of Health Sciences, Kio University, Nara, Japan; ^6^Neurorehabilitation Research Center of Kio University, Nara, Japan; ^7^Department of Neuromodulation and Neurosurgery, Osaka University Graduate School of Medicine, Osaka, Japan; ^8^Department of Rehabilitation Medicine, Nishiyamato Rehabilitation Hospital, Nara, Japan; ^9^Department of Rehabilitation, National Hospital Organization Wakayama Hospital, Wakayama, Japan; ^10^Faculty of Rehabilitation, Kawasaki University of Medical Welfare, Okayama, Japan

**Keywords:** stochastic resonance, galvanic vestibular stimulation, arterial pressure, heart rate variability, RR interval variability, whole-body tilting

## Abstract

**Objective:**

Noisy galvanic vestibular stimulation (nGVS) is often used to improve postural stability in disorders, such as neurorehabilitation montage. For the safe use of nGVS, we investigated whether arterial pressure (AP) and heart rate vary during static supine and slow whole-body tilt with random nGVS (0.4 mA, 0.1–640 Hz, gaussian distribution) in a healthy elderly population.

**Methods:**

This study was conducted with a double-blind, sham-controlled, cross-over design. Seventeen healthy older adults were recruited. They were asked to maintain a static supine position on a bed for 10 min, and the bed was tilted up (TU) to 70 degrees within 30 s. After maintaining this position for 3 min, the bed was passively tilted down (TD) within 30 s. Real-nGVS or sham-nGVS was applied from 4 to 15 min. The time course of mean arterial pressure (MAP) and RR interval variability (RRIV) were analyzed to estimate the autonomic nervous activity.

**Result:**

nGVS and/or time, including pre-/post-event (nGVS-start, TU, and TD), had no impact on MAP and RRIV-related parameters. Further, there was no evidence supporting the argument that nGVS induces pain, vertigo/dizziness, and uncomfortable feeling.

**Conclusion:**

nGVS may not affect the AP and RRIV during static position and whole-body tilting or cause pain, vertigo/dizziness, and discomfort in the elderly.

## Introduction

Non-invasive brain and cranial nerve stimulations are useful treatment modalities for disorders, such as neurorehabilitation montage (Adair et al., 2020). Galvanic vestibular stimulation (GVS) (Fitzpatrick and Day, 2004) is often used both to test vestibular function and as a treatment (Lopez, 2016; Sluydts et al., 2020). It has recently been reported that transcranial stochastic galvanic stimulation of the vestibular nerve improves the stability of posture in elderly people (Fujimoto et al., 2016) and patients with a vestibular disorder (Fujimoto et al., 2018). Noisy GVS (nGVS) (Wuehr et al., 2017) supposedly improves body balance by modulating the threshold of motor response through vestibular input (Fujimoto et al., 2016, 2018; Inukai et al., 2018b). The vestibular system contributes to autonomic regulation (Yates et al., 2014; McCall et al., 2017), and sinusoidal GVS can impact the blood pressure (BP) (Tanaka et al., 2012) and heart rate (HR) variability (Tanaka et al., 2014). A ballistic head-up tilt in the spine capable of activating the vestibular complex system can induce BP and HR variability, and this effect is further facilitated by sinusoidal GVS (Tanaka et al., 2014), indicating that sinusoidal GVS facilitates the vestibular autonomic reflex (Radtke et al., 2003). A strong square wave pulse GVS given to a conscious rat was observed to obscure the arterial pressure (AP) response (Abe et al., 2008). Furthermore, electrical stimulation over and around the ear might stimulate the vagus nerve (Adair et al., 2020) and induce changes in BP and HR-variability (Balasubramanian et al., 2017). However, there is no concrete evidence to prove that nGVS, which is used to improve body balance in the elderly population, can affect BP, HR, and HR variability in the elderly population. Therefore, in this study, we investigated the effect of nGVS on BP and HR, including HR variability, in a healthy elderly population in a static supine position to obtain evidence for ensuring safety when using nGVS.

The ability of nGVS to improve stability and body balance depends on the instability in the upright standing posture before the stimulation (Inukai et al., 2018b), indicating that the effect of nGVS may change depending on the position of the head or/and whole-body movement. The modulation of BP and HR-variability during whole-body movement, including the change in the head direction in response to gravity, by nGVS should be probed (Tanaka et al., 2014). However, both BP and HR are modulated during/after a voluntary movement (Lawrence et al., 2015). Therefore, we used a moving bed, which can change the angle of the whole-body passively, to investigate the effect of nGVS on BP and HR-variability during whole-body movement while preventing voluntary movement.

The primary vestibular nerve projects to the rostral ventrolateral medulla in the brain stem, and the activation of the vestibular nerve by head movement induces the vestibular autonomic reflex, which majorly includes modulation of BP and HR (Yates et al., 1995). A previous study in cats revealed that whole-body tilting up (from a horizontal level to 60°) induces a 30% change in blood flow volume in the leg about 20 s after the tilt-up (TU) (Yavorcik et al., 2009). This finding indicates that BP and HR modulation may occur 20 s after stimulating positional change for the whole-body. Therefore, in this study, we measured and analyzed BP and HR for 20 s or more before/after the event (TU, tilt-down (TD), and GVS-onset).

BP is modulated by autonomic nerve activity (Guyenet, 2006). Autonomic nerve activity can be estimated by measuring the variation in RR-intervals available from electrocardiography (ECG) (Kon et al., 2006; Kuwahata et al., 2011). The coefficient of variation of RR intervals (CVRR) is determined by dividing the standard deviation of RR intervals by the mean RR interval (Kon et al., 2006), and it is considered to reflect the activity of the sympathetic nerve (Kuwahata et al., 2011). A previous study performed a power spectral analysis of the RR-interval (Pagani et al., 1986) to elucidate the effect of head-up tilting on autonomic nerve activity (Tanaka et al., 2014). The high frequency (HF) component was considered to reflect the activity of the parasympathetic nerve; the low frequency (LF) component reflected the activity of both sympathetic and parasympathetic, and the LF/HF ratio reflected the activity of the sympathetic nerve (Pagani et al., 1986). Based on these findings, the CVRR, LF, HF, and LF/HF ratio were calculated to estimate the activity of the sympathetic and/or parasympathetic nerve in nGVS, TU, and TD.

Bilateral bipolar square wave direct current GVS may induce slight pain at the site of electrode placement after stimulation with 1.5 mA intensity in healthy and stroke patients (Utz et al., 2011; Dlugaiczyk et al., 2019). Further, GVS may induce a sensation of vertigo/dizziness (Dlugaiczyk et al., 2019) and nausea (Quinn et al., 2015), and these sensations might appear in a patient with a vestibular disorder (Chen et al., 2020). Therefore, data on the degree of pain, vertigo/dizziness, and uncomfortable feeling were collected from all participants after examination using the nGVS montage.

Based on the above background knowledge, we aimed to probe the hypothesis that nGVS does not modulate AP and HR variability in elderly people during static and dynamic postural change without pain, vertigo/dizziness, and discomfort. We analyzed the time course of the mean arterial pressure (MAP), HR, CVRR, LF, HF, and LF/HF in Sham-/Real-nGVS, TU, and TD in a healthy elderly population. Further, the degree of pain, vertigo/dizziness, and level of discomfort were analyzed for each stimulation condition.

## Materials and Methods

### Participants

Before conducting the experiments, the appropriate sample size was estimated by power analysis using software G^∗^power (Version 3.1.9.4) (Faul et al., 2007) for a two-way analysis of variance (ANOVA). The effect size f was set to 0.4, alpha error probability to 0.05, beta error probability to 0.95, number of groups to 2, and number of measurements to 6. The calculated total sample size was 12; therefore, 18 subjects were recruited with an anticipation of a 30% dropout. One subject was precluded from the experimental analysis because the MAP could not be measured.

All participants were recruited through the Daito silver human resource center in Daito city. The inclusion criteria were: (1) age > 65 years, (2) no history of cardiovascular or otolaryngological disease, (3) no history of neurological disease, including epilepsy, and (4) ability to understand and agree to the contents of this experiment. Seventeen healthy elderly people (13 males, mean age: 74.5 ± 6.0 years, mean height: 160.7 ± 6.5 cm, mean weight: 58.8 ± 8.3 kg) participated in the study. During registration, the participants did not report any history of epilepsy or other neurological diseases, which are especially related to the vestibular system. The Ethics Committee of Shijonawate Gakuen University approved the experimental procedures (Approval Code: 19-5), and the study was conducted according to the principles and guidelines of the Declaration of Helsinki (World Medical, 2013). All participants provided written informed consent.

### Experimental Procedure

This was a double-blinded, sham-controlled, cross-over design study. The participants and assessors were blinded to the nGVS condition, both before and after the experiment. The participants joined a Real-nGVS condition trial and a Sham-nGVS condition trial with an interval of more than 3 weeks between the two. The stimulation condition was selected randomly.

**FIGURE 1:**
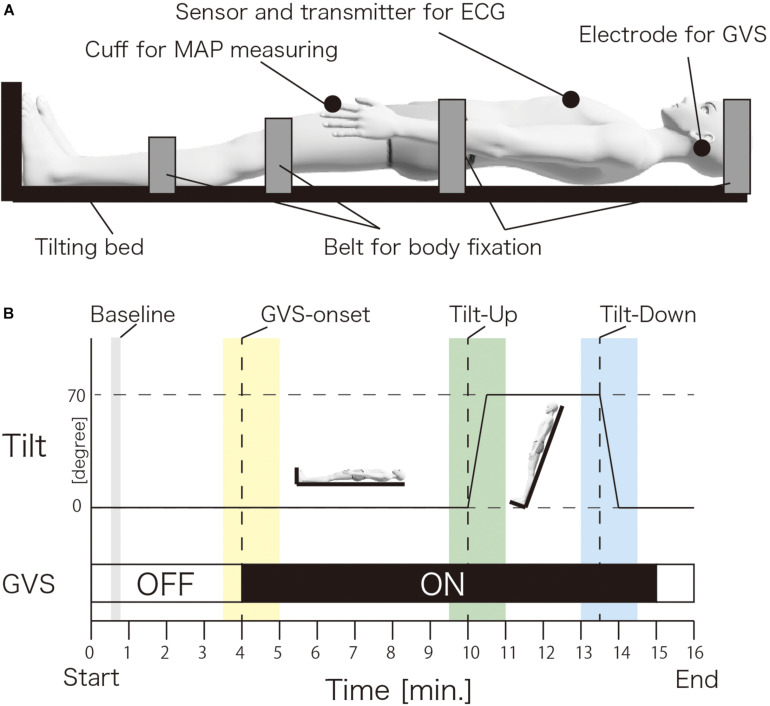
Experimental setup **(A)** and procedure **(B)**. **(A)** The participant was in a supine position on a flatbed. The head, pelvis and both lower limbs were fixed by belts. The electrode for galvanic vestibular stimulation was set to bilateral mastoid processes. The sensor and transmitter for the electrocardiography were set around the center of the chest. A cuff for measuring the arterial pressure was set to the left index finger. **(B)** The left vertical line indicates the degree of tilt. The bottom horizontal line indicates the time. The dotted vertical lines indicate the GVS-onset, Tilt-up start and Tilt-down start. The gray, yellow, green, and blue vertical lines indicate the baseline, GVS-onset, Tilt-up, and Tilt-down measurement time window. The horizontal white line indicates GVS-off and black line indicates GVS-on. MAP; mean arterial pressure, ECG; electrocardiography, GVS; galvanic vestibular stimulation.

Figure 1A shows the experimental setup. The participant was in a supine position on a flatbed in a head-up tilting position (UA-790/795, OGwellness, Japan). The head, pelvis, and both lower limbs of the participants were fixed to the bed using belts. Both feet were in contact with the footplate. A sensor and transmitter for electrocardiography (ECG) were set around the center of the chest. A cuff for measuring the AP was set to the left index finger. The electrode for GVS was set to the bilateral mastoid process.

Figure 1B shows the experimental procedure. The participant was asked to relax with eyes closed during the experiment and was held in a supine position for 10 min. After 10 min, the bed was tilted up from the flat (horizontal) level to 70° in 30 s (TU) (Theodorakis et al., 2003) without announcing the start of TU. This semi-standing position was held for 3 min. After a 3 min hold in this semi-standing position, the bed was tilted down to a flat (horizontal) level within 30 s (TD). The Real-nGVS or Sham-nGVS was delivered from the 4th min to the 15th min. The AP and ECG were recorded during the experiment.

After experimenting in both nGVS conditions, the degree of pain, vertigo/dizziness, and discomfort around the ear were measured with a Visual Analog Scale (VAS) (Heller et al., 2016). In case of no sensation, the participant was asked to report 0 mm. For maximum sensation, the participant was asked to report 100 mm. For a middle-grade sensation between no sensation and the maximum, the participant was asked to report 50 mm.

### AP Measurement

AP was continuously measured using Finometer MIDI (Finapress Medical Systems B.V., Netherlands) and BeatScope Easy (v02.10 build 004, Finapress Medical Systems B.V., Netherlands) connected to a personal computer. This medical device and software are used to diagnose orthostatic dysregulation (Romero-Ortuno et al., 2013) in Japan.

Figure 1A shows the setup for AP measurement. The upper limbs were held on the side of the body, and the cuff and sensor were attached to the distal phalanx of the left index finger (Jagomagi et al., 2010). The sampling rate was set to 0.5 Hz. The MAP was calculated using a built-in-formula as follows: MAP = (systolic AP + 2^∗^diastolic AP)/3. For the time-course analysis of MAP (Leonetti et al., 2004), the mean of MAPs for 10 s was calculated from the start of the event (GVS, TU, and TD) to −20, −10, 10, 20, 30, 40, 50, and 60 s.

### ECG and RR Interval Analysis

We used a real-time heart rate (HR) variability analysis program MemCalc/Bonaly Light (GMS Co., Ltd., Tokyo) and a wireless biometric sensor and transmitter (RF-ECG, transmit frequency: 2.4 GHz, MemCalc, GMS Co., Ltd., Tokyo) to measure the ECG. Two Ag/AgCl electrodes (Blue Sensor EKG Snap Electrode, overall dimensions: 48 × 57 mm, Ambu, Baltorpbakken, Denmark) were attached to the left anterior portion of each subject’s chest, and the ECG signals were wirelessly transmitted to a personal computer.

Based on the ECG data obtained, beat-to-beat RR intervals were linearly interpolated depending on the subjects’ HR followed by resampling at 1.2 Hz to obtain an equidistant time series using the MemCalc/Bonaly Light (GMS Co. Ltd., Tokyo) analysis program. The HR (number of heartbeats in 1 min) was calculated from the RR intervals in each sample. A power spectral analysis was performed within a 30 s time window using the same analysis program, and the power of LF component (0.04–0.15 Hz, LF, s^2^) and HF component (0.15–0.4 Hz, HF, s^2^) was calculated (Pagani et al., 1986). A moving average of the power was calculated and updated every 2 s, and as a representative value, the mean LF power and HF power was calculated for 10 s from the start of the event (GVS, TU and TD) to −20, −10, 10, 20, 30, 40, 50, and 60 s, similar to the MAP for time-course analysis.

The changes in LF and HF were used to measure the changes in sympathetic and parasympathetic nerve activity, respectively. The changes in HF were considered a reflection of the modulation in the sympathetic nerve activity, based on a report that LF/HF reflects the modulation in sympathetic nerve activity (Pagani et al., 1986). A previous study performed a beat-by-beat time-course analysis of LF, HF, and LF/HF and the significant modulation of these parameters at approximately 10 beats and 10 s before and after the intervention (Chouchou et al., 2019). Therefore, a 10 s time window can be considered appropriate for this study to analyze the RR interval.

### nGVS

nGVS was performed as previously reported (Matsugi et al., 2020a,b). nGVS was delivered via Ag/AgCl surface electrodes (Blue Sensor EKG Snap Electrode, overall dimensions: 48 × 57 mm, Ambu, Baltorpbakken, Denmark) affixed to the right and left mastoid processes. A DC-STIMULATOR PLUS (Eldith, NeuroConn GmbH, Ilmenau, Germany) was used to deliver random nGVS to the primary vestibular nerve. For nGVS in the stimulation mode, a random level of current was generated for every sample to be used as “noise” (sample rate, 1280 samples/s) (Moliadze et al., 2012; Inukai et al., 2018b), and the intensity was set at 0.4 mA, which was previously reported as an effective intensity for the elderly (Inukai et al., 2018a). Statistically, the random numbers were normally distributed over time, the probability density followed a Gaussian bell curve, and all coefficients featured a similar size for the frequency spectrum in this mode. A waveform was applied with 99% of the values between –0.5 and + 0.5 mA, with only 1% of the current level within ± 0.51 mA. The stimulation time was set to 660 s without being ramped up and down. For the sham stimulation, direct current stimulation was applied at an intensity of 0 mA (sham-nGVS).

### Statistical Analysis

The MAP/baseline, HR/baseline, CVRR/baseline, LF/baseline, HF/baseline, and LF/HF/baseline were calculated. Next, we discarded the outlier data-points with mean ± 5 times of standard deviation (SD) in MAP, HR, CVRR, LF, HF, and LF/HF in GVS-on, TU, TD in sham and real-nGVS condition.

Bayesian hypothesis test can assist in the interpretation of null results, and this method was used in the standalone analyses (Dienes et al., 2018; Hoekstra et al., 2018; Matsugi et al., 2019; van Ravenzwaaij et al., 2019; Keysers et al., 2020). Bayesian Wilcoxon signed-rank was used to estimate the evidence supporting the hypothesis that the VAS score for pain, vertigo/dizziness, and discomfort is not significantly different. To test the hypothesis that stimulation and time does not affect MAP, HR, CVRR, LF, HF, and LF/HF, a Bayesian Two-way Repeated Measures analysis of variance (ANOVA) (Stimulation ^∗^ Time) was performed while assuming equal distribution, based on a previous study (Matsugi et al., 2019). In case equal distribution was not assumed, we used Bayesian One-way Repeated Measures ANOVA. If parametric one-way ANOVA was used after assumption check for using one-way ANOVA, Kruskal-Wallis test, as a non-parametric test, was applied to test the difference. The alpha level was set to 5% for the assumption test and Kruskal-Wallis test.

For the Bayesian test, posterior odds were corrected for multiple testing by fixing a prior probability that the null hypothesis holds good across all comparisons at 0.5 (Westfall et al., 1997). Statistical analyses were performed using the JASP software (version 0.12.2; University of Amsterdam, Amsterdam, Netherlands) (Team, 2019; Keysers et al., 2020). The most common prior model that was default in the software was selected, based on the methods reported previously (Hoekstra et al., 2018; Matsugi et al., 2019), and r scale fixed effects = 0.5, r scale random effects = 1, and r scale covariates = 0.354 were used.

We estimated the predictive performance of two competing hypotheses: the null hypothesis, wherein stimulation and time had no effect, and the alternative hypothesis wherein stimulation and time had an effect (Hoekstra et al., 2018). The Bayes factor (BF) (Hobbs and Carlin, 2008) allows researchers to quantify the evidence in favor of the null hypothesis (Zaslavsky, 2013; Hoekstra et al., 2018). If BF_10_ is > 3, it is considered that there is more than substantial evidence for accepting the alternative hypothesis (Hoekstra et al., 2018). In contrast, if BF_10_ is < 1, it is believed that there is no evidence for accepting the alternative hypothesis (Hoekstra et al., 2018). Further, if 3 < BF_10_ < 1, it is considered that there exists mixed evidence supporting null and alternative hypotheses.

## Results

Seventeen elderly participants completed all the experiments, and their data were used for the analysis. Before the examination, data from one participant was not included because the MAP could not be measured (see section “Materials and Methods”). No adverse effects necessitating the stoppage of the study were observed during or after all the trials.

Table 1 shows the result of VAS for pain, vertigo/dizziness, and discomfort. Only 1 participant reported very slight vertigo/dizziness (Sham: 7 mm, Real: 2 mm) and discomfort (Sham: 3 mm, Real: 3 mm). The participant reported that these sensations occurred at the start of TU for some seconds in both Sham and Real-nGVS conditions. The BF_10_ for vertigo/dizziness and discomfort were 0.34 and 0.36, respectively. In contrast, a Bayesian Wilcoxon test could not be conducted for pain because all the values were 0 in Sham- and Real-nGVS conditions. This indicates that nGVS was conducted in the subthreshold of sensation.

**TABLE 1:** Visual Analog Scale for pain, vertigo/dizziness, and uncomfortable feeling.

	Sham			Real		
Subject	Pain	Vertigo/dizziness	Uncomfortable feeling	Pain	Vertigo/dizziness	Uncomfortable feeling
1	0	0	0	0	0	0
2	0	0	0	0	0	0
3	0	0	0	0	0	0
4	0	0	0	0	0	0
5	0	0	0	0	0	0
6	0	7	3	0	2	3
7	0	0	0	0	0	0
8	0	0	0	0	0	0
9	0	0	0	0	0	0
10	0	0	0	0	0	0
11	0	0	0	0	0	0
12	0	0	0	0	0	0
13	0	0	0	0	0	0
14	0	0	0	0	0	0
15	0	0	0	0	0	0
16	0	0	0	0	0	0
17	0	0	0	0	0	0

*The unit is in mm.*

**FIGURE 2:**
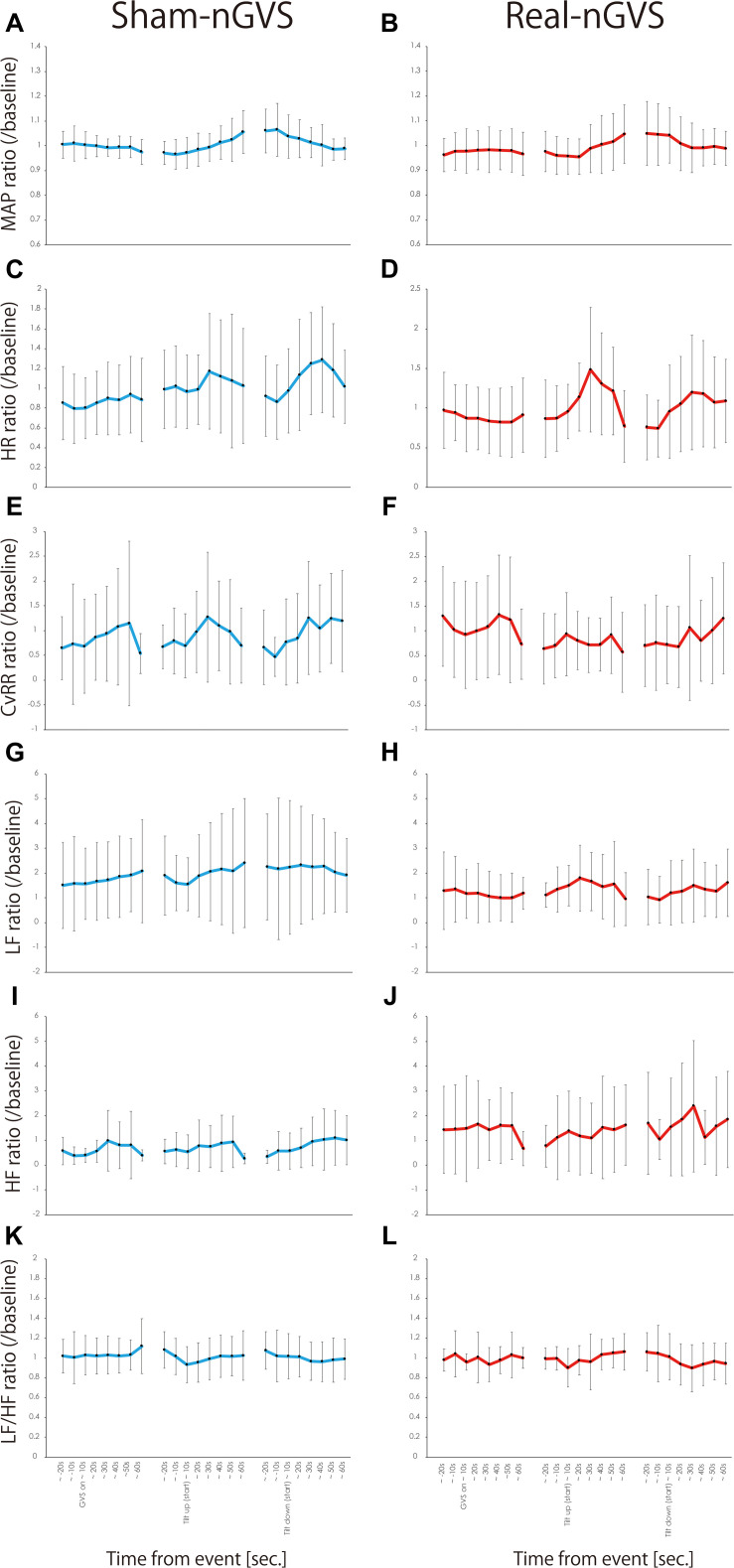
MAP **(A,B)**, HR **(C,D)**, CVRR **(E,F)**, LF **(G,H)**, HF **(I,J)**, and LF/HF **(K,L)** in Sham-nGVS (left line) and Real-nGVS (right line). The circles indicate the mean values per baseline, and error bar indicate standard deviation. MAP, mean arterial pressure; HR, heart rate; CVRR, coefficient of variation in RRI; LF, low-frequency component; HF, high-frequency component.

Figures 2A–L shows the time-course of MAP, HR, CVRR, LF, HF, and LF/HF. The assumption test for two-way ANOVA revealed that all conditions were not distributed equally between the two groups. Bayesian one-way ANOVA could not be applied to LF in TU in Sham condition, HF in TU in sham and real condition, and LF/HF in GVS-on, TU, and TD in sham condition, and TD in real nGVS condition. For these parameters, the Kruskal-Wallis test was applied, and no significant difference was observed between the time. In another condition, Bayesian one-way ANOVA was applied, and BF_10_ was observed to be > 1. These results indicate that there was no change in all parameters between both sham- and real-nGVS conditions. All data and results of the analyses along with code in JASP are available online in data storage^1^.

## Discussion

To probe the hypothesis that nGVS does not modulate AP and HR variability without harmful sensation in a static position and/or dynamic postural change, we analyzed the time-course of MAP, HR, CVRR, LF, HF, and LF/HF immediately before/after GVS-onset, TU, and TD in 17 healthy elderly people. The result of the Bayesian one-way ANOVA and Kruskal-Wallis test showed no evidence supporting the hypothesis that nGVS could change MAP and RR-related parameters in GVS-onset, TU, and TD. Further, the Bayesian Wilcoxon test result showed no evidence supporting the hypothesis that nGVS induces pain, vertigo/dizziness, and discomfort. These findings indicate that stochastic noisy electrical stimulation to the vestibular nerve may not impact the AP, HR, and HR variability without pain, vertigo/dizziness, and a sensation of discomfort.

The potential effects of nGVS has been explored through basic research on noise stimulation. The stochastic resonance phenomenon of enhanced non-linear response to an input signal has been reported to be involved in the effects of nGVS (Douglass et al., 1993). In the vestibular system, a particular level of mechanical noise on the semicircular canals can improve the performance of the vestibular system in peripheral sensory processing (Flores et al., 2016). nGVS-induced modulation of the threshold of the vestibulospinal response (Matsugi et al., 2020a) is thought to contribute to body sway changes (Matsugi et al., 2020b; Sprenger et al., 2020). The vestibular system contributes to autonomic regulation (Yates et al., 2014; McCall et al., 2017); therefore, nGVS might modulate AP and HR variability. However, our study results indicate nGVS has no effect on AP and HR variability during the static and dynamic postural change in older adults.

We observed that nGVS with 0.4 mA intensity has no effect on AP and HR variability without pain, vertigo/dizziness, and uncomfortable feeling in elderly people. A previous study reported that nGVS with 0.4 mA intensity can improve postural stability of community-dwelling elderly people without serious harm (Inukai et al., 2018a). Both of these findings are consistent with each other, supporting the fact that nGVS exerts no harmful effect. Sinusoidal GVS reportedly alters the RR interval variability in young adults and modulates the vestibular autonomic response of AP (Tanaka et al., 2012, 2014). Further, previous studies have reported that GVS may induce a sensation of vertigo/dizziness (Dlugaiczyk et al., 2019) and nausea (Quinn et al., 2015). One possible reason for the difference in AP response and harmful sensation may originate from the stimulation type, which is an electrical stimulation with a noisy and sinusoidal waveform. Another possible reason is the difference in intensity (that is 2 and 0.4 mA). Non-invasive brain stimulation effect depends on the electrical stimulation pattern (Finisguerra et al., 2019). The nGVS effect on AP and HR variability may depend on the stimulation pattern.

In this study, the pain was not induced by nGVS in any participant. A previous study reported that the pain threshold for direct current electrical stimulation is approximately 4 mA in humans (Lobel et al., 1998). A square-wave pulse GVS of 3 mA for 200 ms with an electrode over the bilateral mastoid process, similar to that in this study, did not induce pain (Matsugi et al., 2017; Okada et al., 2018), and continuous direct current GVS of 1 mA induced a slight sensation in about 25% of young adult participants (Nakamura et al., 2020). On the contrary, nGVS of 1 mA intensity using the same stimulator as in our study did not induce pain in 30 young adults in a static prone position (Matsugi et al., 2020a) and 17 young adults in a static standing position (Matsugi et al., 2020b). Therefore, an intensity of 0.4 mA for nGVS might be below the threshold of sensation, and based on these findings, we believe that nGVS at 0.4 mA cannot induce pain in the elderly population. However, one female participant suffered from slight vertigo/dizziness and discomfort. However, this participant reported that this sensation was felt in both examinations and only a few seconds after the TU started. Therefore, vertigo/dizziness and a sensation of discomfort may not be associated with stimulation and are possibly caused by the head movement accompanying the TU. In vertigo/dizziness induced by the change in head position, the rate of occurrence in women is about 2 times more than that in men, and the prevalence of this condition in the elderly population is about 10% (von Brevern et al., 2007). Perhaps this female participant had an undiagnosed, mild Benign Positional Paroxysmal Vertigo (Mandala et al., 2019).

There were some limitations in this study. We observed no change in all parameters immediately before/after the event; however, we cannot deny the possibility that there was modulation at other times, such as the time from 60 s after the GVS to just before the TU. Next, the effect of nGVS on the balance depends on the postural instability before stimulation (Inukai et al., 2018b). However, we did not examine the effect of nGVS on postural stability in these participants. Therefore, we cannot exclude the possibility that not all participants responded to nGVS. Further studies are needed to correlate the degree of effect of nGVS on postural stability, AP, and HR. Moreover, there was no change in MAP in the sham-nGVS condition in TU in our results; therefore, we cannot reject the possibility that nGVS modulates AP and HR variability in elderly people with cardiocirculatory disorders, such as orthostatic hypotension, arrhythmia, and heart failure. In line with the results, we believe that our findings are limited to a healthy elderly population. Another limitation is that our TU method is acceptable to test for orthostatic hypotension; however, the speed of TU may weakly induce the modulation of AP in healthy elderly people. There is a possibility that the effect of nGVS on AP and HR variability may manifest with faster TU and TD.

Regarding the clinical significance of this study, our result provides evidence for the safety of using nGVS in elderly people. While previous studies report the effectiveness of nGVS (Fujimoto et al., 2016, 2018, 2019; Inukai et al., 2018a,b), they provide no evidence on its impact on AP and HR variability. In future studies, the effect of nGVS on AP and HR variability in patients with cardiocirculatory disorders, such as orthostatic hypotension, arrhythmia, and heart failure, should be tested further to ensure safety.

In conclusion, we observed that stochastic electrical stimulation of the vestibular nerve does not affect AP and HR variability in healthy elderly population during static position and dynamic passive postural change, without any harmful sensation. These findings may provide evidence for the safety of nGVS use in elderly people to improve postural stability.

## Data Availability Statement

The datasets presented in this study can be found in online repositories. The names of the repository/repositories and accession number(s) can be found below: http://dx.doi.org/10.17632/8gx48tmvjr.2.

## Ethics Statement

The studies involving human participants were reviewed and approved by the ethics committee of the Shijonawate Gakuen University. The patients/participants provided their written informed consent to participate in this study.

## Author Contributions

AM, KN, and TS: conceptualization. AM, TS, YO, NM, JN, SD, KO, and YT: data curation. AM and KN: formal analysis and methodology. AM, KN, TS, YO, and KN: funding acquisition. KN: resources, software, and supervision. AM, TS, NM, JN, SD, KO, and YT: validation. AM: visualization and writing—original draft. AM, KN, TS, YO, NM, JN, SD, KO, and YT: writing—review and editing. All authors contributed to the article and approved the submitted version.

## Conflict of Interest

The authors declare that the research was conducted in the absence of any commercial or financial relationships that could be construed as a potential conflict of interest.

## Footnotes

1 http://dx.doi.org/10.17632/8gx48tmvjr.2
